# Precision rehabilitation for cerebral palsy will require robust measures of motor control development

**DOI:** 10.1111/dmcn.16460

**Published:** 2025-08-07

**Authors:** Laura A. Prosser

**Affiliations:** ^1^ Division of Rehabilitation Medicine, The Children's Hospital of Philadelphia Philadelphia PA USA; ^2^ Department of Pediatrics University of Pennsylvania Philadelphia PA USA

## Abstract

**This commentary is on the original article by Staudt et al. on pages** 211–217 **of this issue.**

Despite wide heterogeneity in the severity and phenotype of cerebral palsy (CP), universally, the primary deficit is the poor development of motor control.^1^ In fact, impairment in selective motor control (SMC) has repeatedly been a better predictor of function than spasticity, muscle weakness, or joint contracture.^2^ Yet the development of motor control is not routinely measured due to the scarcity of measurement tools.

Designed for children with or at risk for CP between 3 months and 4 years of age, the Mini‐SCALE^3^ advances assessment of lower limb SMC in young children, bridging the age gap between the BabyOSCAR^4^ and SCALE.^5^ This age range is particularly important because motor control impairments in young children with CP may be more amenable to treatment than in older children, before the neural pathways that support movement are established and less flexible.

Staudt et al.'s study provides evidence of clinical feasibility and of content and construct validity for the Mini‐SCALE.^3^ Regrettably, it does not include reliability data. Scores from younger infants and in the grade of ‘impaired’ (1 point) may demonstrate lower reliability than from older children and in the ‘normal’ (2 points) and ‘unable’ (zero points) grades. It also appears that most children who were at risk for CP, and some with diagnosed CP, scored within the range observed in the typically developing cohort, suggesting that Mini‐SCALE may identify severe–moderate cases but miss cases of mild CP. Thorough investigations of reliability and predictive validity by age, joint, and rank score are needed to guide interpretation related to early detection of risk for CP in the context of developmental maturation.

Difficulty with hip joint scoring in infants under 9 months of age presents a significant limitation. The authors propose interpreting a total score of 8 (rather than 10) as normal for this age (disregarding the hip score but not omitting the item from testing). It is unclear how a total score of 8 or 9 should be interpreted in children under 9 months if any joint other than the hip scores <2, as might be expected often in children with mild bilateral (diplegia) or unilateral (hemiplegia) CP. From a theoretical perspective, the authors suggest that muscle weakness, not poor SMC, is the primary factor in hip scores <2 in younger infants with typical development, which implies inadequate construct validity of this item. This concern could be addressed in a future revision of the hip item—perhaps to allow a partially flexed knee posture so long as the knee joint is not moving during hip movement (given the reported challenges also with testing the hip in side‐lying). These questions underscore the need for longitudinal testing in an age‐stratified sample.

The Mini‐SCALE is a commendable first step addressing the need for clinically feasible measures of lower limb SMC that can be administered in infants and young children. It has potential to guide the selection of early intervention strategies, based on the general degree of SMC impairment in each lower limb. However, like other ordinal scales (e.g. the SCALE and Gross Motor Function Classification System), it is not designed to be sensitive to change over time or after intervention. Tools that offer greater precision on the type, location, and magnitude of SMC impairment have potential to complement these ordinal clinical scales to improve the prediction of CP subtyping (including non‐spastic types and severity level) and to serve as sensitive endpoints for clinical trials—both critical factors in moving the field toward precision rehabilitation.

With advances in the early detection of CP, tools to measure SMC development in infants and young children are vital to design effective interventions and to evaluate their efficacy in select subgroups during the critical neuroplastic period for motor control.

## FUNDING INFORMATION

There was no financial sponsor of this work.

## CONFLICT OF INTEREST STATEMENT

The author has stated that they had no interests which might be perceived as posing a conflict or bias.

## Data Availability

Data sharing is not applicable as no datasets were generated or analysed in this work.
